# Co- transplantation of Bone Marrow Stromal Cells with Schwann Cells Evokes Mechanical Allodynia in the Contusion Model of Spinal Cord Injury in Rats

**Published:** 2011-12-22

**Authors:** Bagher Pourheydar, Mohammad Taghi Joghataei, Mehrdad Bakhtiari, Mehdi Mehdizadeh, Zahra Yekta, Norooz Najafzadeh

**Affiliations:** 1. Department of Anatomy, Faculty of Medicine, Urmia University of Medical Sciences, Urmia, Iran; 2. Department of Anatomy, Faculty of Medicine, Tehran University of Medical Sciences, Tehran, Iran; 3. Department of Epidemiology, Faculty of Medicine, Urmia University of Medical Sciences, Urmia, Iran; 4. Department of Anatomy, Faculty of Medicine, Ardabil University of Medical Sciences, Ardabil, Iran

**Keywords:** Cell Transplantation, Stem Cell, Spinal Cord Injuries, Allodynia

## Abstract

**Objective:**

Several studies have shown that, although transplantation of neural stem cells into the contusion model of spinal cord injury (SCI) promotes locomotor function and improves functional recovery, it induces a painful response, Allodynia. Different studies indicate that bone marrow stromal cells (BMSCs) and Schwann cells (SCs) can improve locomotor recovery when transplanted into the injured rat spinal cord. Since these cells are commonly used in cell therapy, we investigated whether co-transplantation of these cells leads to the development of Allodynia.

**Materials and Methods:**

In this experimental research, the contusion model of SCI was induced by laminectomy at the T8-T9 level of the spinal cord in adult female wistar rats (n=40) weighting (250-300g) using the New York University Device. BMSCs and SCs were cultured and prelabeled with 5-bromo-2-deoxyuridine (BrdU) and 1,1'-dioctadecyl-3,3,3',3'-tetramethylindocarbocyanine perchlorate (DiI) respectively. The rats were divided into five groups of 8 including: a control group (laminectomy only), three experimental groups (BMSC, SC and Co-transplant) and a sham group. The experimental groups received BMSCs, SCs, and BMSCs and SCs respectively by intraspinal injection 7 days after injury and the sham group received serum only. Locomotion was assessed using Basso, Beattie and Bresnahan (BBB) test and Allodynia by the withdrawal threshold test using Von Frey Filaments at 1, 7, 14, 21, 28, 35, 42, 49 and 56 days after SCI. The statistical comparisons between groups were carried out by using repeated measures analysis of variances (ANOVA).

**Results:**

Significant differences were observed in BBB scores in the Co- transplant group compared to the BMSC and SC groups (p< 0.05). There were also significant differences in the withdrawal threshold means between animals in the sham group and the BMSC, SC and the Co-transplant groups (p<0.05).BBB scores and withdrawal threshold means showed that co-transplation improved functioning but greater Allodynia compared to the other experimental groups.

**Conclusion:**

The present study has shown that, although transplantation of BMSCs, SCs and a combination of these cells into the injured rat spinal cord can improve functional recovery, it leads to the development of mechanical Allodynia. This finding indicates that strategies to reduce Allodynia in cell transplantation studies are required.

## introduction

Spinal cord injury (SCI) is one of the most disabling diseases which leads to neural tissue damage, significant sensorimotor deficits and disruption of autonomic nervous system control in areas caudal to the injury site (1). Injury to the spinal cord can also lead to development of chronic pain conditions (2) such as Allodynia.

Allodynia is an abnormal pain syndrome in which innocuous stimuli gain the ability to produce pain. Epidemiological studies have reported that more than 64% of patients with spinal cord injuries suffer from chronic pain syndromes (3).

Allodynia has various physical and psychological effects on patients which compromise their quality of life (4) and they have a poor ability to work. Two mechanisms have been proposed to explain the development of Allodynia. One hypothesis involves the pathological loss of GABAergic interneurons in the superficial dorsal horn (lamina Ι-ΙΙΙ which is associated with nociception) after SCI (5). The second mechanism is that stem cells, such as neural stem cells (NSCs), transplanted into the injured spinal cord differentiate mainly into astrocytes (6) which provide nerve growth factor (NGF) (7). NGF causes aberrant axonal sprouting in neurons of the dorsal horn which is associated with nociception and this leads to Allodynia (8).

Different researchers have demonstrated that transplantation of stem cells like bone marrow stromal cells (BMSCs) (9), Schwann cells (SCs) (10), neural stem cells (NSCs) (11), or Olfactory bulb ensheathing cells (OEC) (12) into the injured spinal cord improves locomotor recovery . Other studies have shown that transplantation of NSCs improves functional recovery but induces neuropathic pain (mechanical Allodynia) (13). Since BMSCs (14) and SCs (15) are commonly used in cell therapy studies, we sought to investigate whether co-transplantation of these cells into the injured spinal cord could improve functional recovery and whether co-transplantation would induce Allodynia.

## Materials and Methods

### Animals

In this experimental research, adult female Wistar rats (n=40) (Pasteur Institute, Tehran) weighting (250-300g) were used. All procedures in this study, including the use of animals, were approved by the Research Council of Tehran University of Medical Sciences (Tehran, Iran), Ethics Committee on Animal Experiments whose guidelines are in agreement with those of the National Institutes of Health for the use of live animals.

### Bone marrow stromal cell isolation

Bone marrow was isolated in sterile conditions from 8 weeks-old male Sprague Dawley rats weighting (250-300g) as described in detail by Azizi et al. (16). Briefly, rats were killed with an overdose of pentobarbital and the tibia and femur were dissected out, both ends of the bones were cut off and marrow was flushed out with 5ml α‒MEM (Sigma, Germany) with a 25-gauge needle. The suspension was centrifuged at 800 rpm for 5minutes and the supernatant was removed. The marrow cells were suspended with 10ml of α‒MEM and cultured in α‒MEM supplemented with 10% fetal bovine serum (FBS), 2ml glutamine, penicillin (100 U/ml, Sigma, Germany) and streptomycin (100µg/ml Sigma, Germany).

After 48 hours, the non-adherent cells were removed by replacing the medium, when the adherent cells had grown to 80% confluency they were removed by incubation in a solution containing 0.25% trypsin and 1 mM EDTA (Sigma, Germany) at 37℃ for 5 minutes and passaged. BMSCs were subcultured 4 times in this way.

### Labeling

The cells were labeled with bromodeoxyuridine (Brdu) at a concentration of 3µg/ml which was added to the incubation medium 3 days prior to transplantation.

### SC isolation

SCs were obtained from the sciatic nerves of adult female wistar rats (10 weeks old) weighting (250-300g), as described by Morrissey et al. (17). Briefly, the rat was anesthetized with a combination of ketamine (80 m/kg) and xylazine (10 mg/ kg). The left sciatic nerve of the animal was exposed and transected at the greater sciatic notch to allow Wallerian degeneration to occur. After 7 days the animal was killed with an overdose of pentobarbital and 20 mm of the distal segment of transected nerve was resected, and placed in a dish containing DMEM (Sigma, Germany).

Under sterile conditions and using a dissecting microscope the epineuruim was removed with a fine forceps, then the nerve washed 3 times with PBS and transferred to a dish containing 10% FBS (Sigma, Germany) and 1.25 IU/ml dispase (Sigma, Germany), 0.05% collagenase type IA (Sigma, Germany) and incubated for 3 hours. Then the cell suspension was centrifuged at 800 rpm for 5minutes, the supernatant was discarded and the pellet was washed with medium and resuspended in DMEM supplemented with 10% FBS, 100 U/ ml penicillin and 100µg/ml streptomycin (Sigma, Germany).

The cell suspension was counted using a hemocytometer and placed in 25cm^3^ flasks and incubated in 5% CO_2_ at 37℃ in medium containing 10% FBS. After 2 days, non-adherent cells were removed by replacing the medium and the adherent cells were allowed to reach to confluent state. Once the adherent cells had reached 80% confluency the medium was replaced with 0.25% trypsin and 1mM EDTA (Sigma, Germany) and incubated at 37℃ for 5minutes

Cell dissociation from the substrate was monitored using an inverted microscope (Olympus 1×70, Japan) until the maximum amount of cells had been lifted. The cell suspension was transferred into a 15ml tube and 10ml PBS was added. After centrifugation the supernatant was removed and replaced with complete medium, and transferred to two 25cm^3^ flasks. The cells were subcultured once a week.

### Schwann cell purification

**Fig 1:**
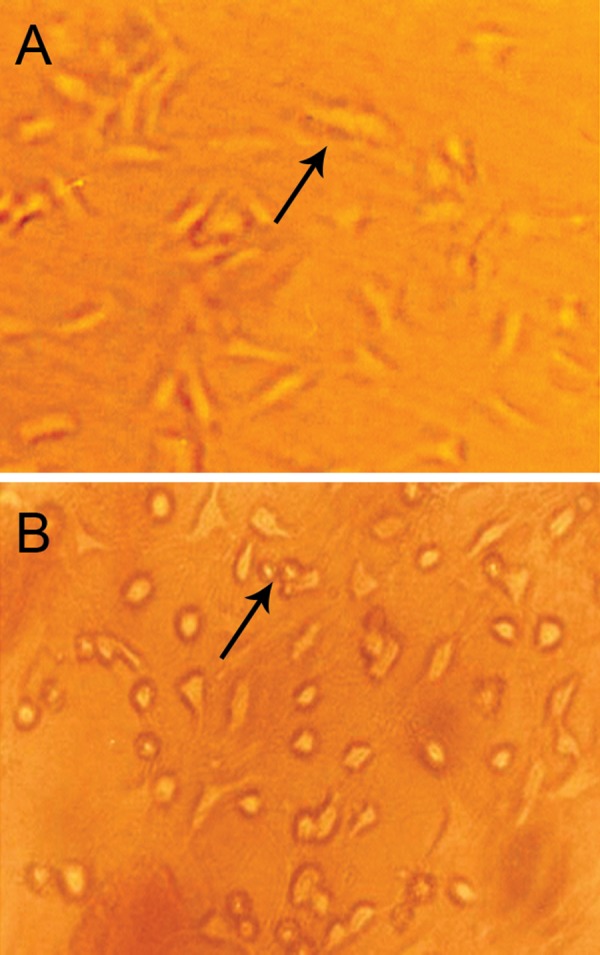
Schwann cells and bone marrow stromal cells in culture. (A) Schwann cells (×200) and (B) bone marrow stromal cells are shown in late stage of subculture (× 200)

After SCs have been enzymatically stripped from the sciatic nerve by incubation with collagenase and dispase, a considerable population of fibroblasts remains within the preparation. For removal of these fibroblasts from the SC culture, the culture was incubated with the antimitotic cytosine arabinoside (Ara-c, 1µl/ml , 5mM, Sigma ,Germany) 24 hours after the cells was first isolated (18) as, after 5 to 7 days, Ara-c will remove the majority of fibroblasts (Fig 1A)

### Schwann cell amplification

SCs cultured alone in vitro divide extremely slowly, but may be stimulated to proliferation by some mitogens. To stimulate the proliferation of SCs in this case the culture was incubated with mitogen Forskolin (2µM, Sigma, Germany) (19).

### Schwann cell labeling

The cells were labeled by the fluorescent lipophilic tracer 1, 1'- dioctadecyl-3, 3, 3', 3' tetramethylindocarbocyanin perchlorate (DiI, Sigma, Germany) prior to transplantation. For labeling, the cells were resuspended at 1×10_6_ cells/ml in α‒MEM, and DiI was added at 5µl/ml in α‒MEM. After incubation for 20 minutes at 37℃ with 5% humidified CO_2_, the cells were centrifuged for 5minutes and washed twice with PBS and were then resuspended in phosphate buffer saline (PBS) for transplantation (20).

### Spinal cord injury model

Female adult wistar rats weighting (250-300g) were used for SCI model. The animals were anesthetized using intraperitoneal ketamine (80mg/ kg) and xylazine (10 mg/kg). Rats were placed prone on an operating table covered with a warming blanket. After shaving the mid-thoracic region and prepping with Betadine, an incision was made over the mid-thoracic region. Laminectomy was performed at the (T8-T9) level of the spinal cord. A standard spinal cord contusion was made using the New York University (NYU) weightdrop device.

A metal rod 10 g in weight and 2 mm in diameter was dropped from a height of 12.5 mm onto the exposed spinal cord at the T8 level to cause moderate contusion. Then the wound was closed and postoperative care included manual bladder expression 2 times a day, administration of Ringer's solution to avoid dehydration (2 ml, ip, after surgery) and administration of gentamicin (0.8mg/100g, ip) during the week after surgery. Analgesia was achieved using buprenorphine (0.1 mg/kg) for 2 days after surgery. Passive mobilization of the hind legs for 15 minutes was undertaken every day for a week after surgery.

### Histology

Four animals were killed 4 weeks after spinal cord injury. Animals were deeply anesthetized with sodium pentobarbital (100 mg/kg, i.p) and were transcardially perfused with 4% paraformaldehyde in 0.1 mol/L PBS, pH=7.4. Tissues were cryoprotected in 30% sucrose overnight and a 1.5 cm segment of spinal cord containing the zone of the injury was removed, embedded in cryo-preservation medium (OCT) and cut transversely into 8µm serial sections. Sections were stained with Cresyl Violet Staining for the examination of general morphology.

### Transplantation procedure

The rats were randomly divided into 5 groups: 1. control group (n=8) in which only a laminectomy was performed; 2. sham group (n=8) in which serum was administered by intraspinal injection; 3.

BMSC group (n=8) which received (3×105 BMSCs) by intraspinal injection; 4. SCs group (n=8) which received (3×10^5^ SCs) by intraspinal injection; 5. co-transplant group (n=8) which received (3×10^5^ BMSCs and SCs) in the same way.

BMSCs were resuspended at a concentration of 30000 cells per µl. Seven days after SCI rats were anesthetized, the contusion site was re-exposed and the cell transplantation was performed drawing the suspension of BMSCs into a Hamilton syringe.

The Hamilton syringe was attached to a microinjector (model 780310) through a customized needle (110µm internal diameter) with a sterile 30- gauge needle. A small hole was made in the dura at the injection site. Then the customized needle was inserted into the spinal cord at the midline to a depth of 1 to 1.5 mm. 0µl of the cell suspension was then injected over 2 minutes. at a distance of 1mm rostrally and then 1 mm caudally from the site of injury. The needle was left in place for 2 minutes after injection before withdrawal to minimize cell leakage.

After each transplantation session, 1 sample of BMSCs and SCs from the Hamilton syringe was mounted onto a slide and stained with trypan blue to assess cell viability. The SCs and the combination of BMSCs and SCs were transplanted into the spinal cord in the same manner. The sham group had serum 'transplanted' in the same way.

### Behavioral assessment (locomotor function)

Locomotor activity was evaluated over a period of 5 minutes using the open-field walking test. One animal at a time was allowed to move freely inside a circular plastic tray (90 cm diameter × 24 cm wall height).

Two independent examiners observed the hind limb movements of the rat and scored the locomotor function according to the Basso Beattie and Bresnahan scale (BBB scale) that ranges from 0 (paralysis) to 21 points (normal gait). The final score for each animal was the mean value of both examiners. During the open field activity the animals were also video monitored with a digital camera. Functional tests were performed before the injury and transplantation and weekly for 8 weeks after transplantation.

### Behavioral assessment (mechanical allodynia)

Mechanical sensitivity in the rats hind paws was assessed using Von Frey Filaments before and weekly for 8 weeks post transplantation, as described by Pitcher et al .(21). The withdrawal threshold hair test was performed by the same investigator for all animals at all time points and another investigator observed the test procedure to ensure consistency. The investigators were blinded to the treatment.

The platform on which the test was performed consists of a transparent plastic box. This box has 1.5 mm diameter holes in its base through which Von Frey Filaments were applied to the plantar surface of the animal٫s hind paws. Filaments consist of hairs ranging from 0.008 to 300g which were applied to determine the hind paw withdrawal threshold.

The animals were placed in the testing box for 15minutes before testing for habituation. All 4 paws were in contact with the platform. After applying the Von Frey Filaments in ascending order, every complete removal of the hind paw from the platform was considered as a withdrawal response. The test was performed 5 times with 5 second intervals. The force of filaments caused hind paw withdrawal and this force in grams demonstrated the paw withdrawal threshold for 4 of 5 consecutive applications.

When the paw withdrawal threshold had been determined for the Right hind paw, the procedure was repeated for the Left hind paw.

### Immunohistochemistry

Rats were sacrificed 8 weeks after transplantation and were deeply anesthetized with sodium pentobarbital (100mg/kg. ip) and were transcardially perfused with 4% paraformaldehyde (0.1 M phosphate buffer, pH =7.4).

Tissue was cryoprotected in 30% sucrose overnight and a segment of the spinal cord 1 cm in length from the injury site was removed, embedded in OCT and cryosectioned transversally into 8µm serial sections.

### Brdu immunohistochemistry

BMSCs which had been labeled with Brdu prior to transplantation were identified according to the following procedure (22). The sections were incubated in 50% Formamide (Merck, Germany), 2× SSC (Standard Sodium Citrate: 0.3M NaCl and 0.03 M sodium citrate) at 65℃ for 2hours, washed for 10 min with 2× SSC at room temperature then incubated in 2N HCl (Merck, Germany) at 37℃ for 30 minutes. They were then rinsed in 0.1 M boric acid (Merck Germany) for 10 minutes, washed in PBS and incubated with Mouse anti- Brdu monoclonal antibody (Sigma, Germany.) at 4℃ overnight.

After rinsing 3 times in PBS for 10min, they were incubated again overnight in the dark at 4℃ with fluorescein isothiocyanate (FITC) conjugated secondary antibody (1:100) (19). They were then washed in PBS, covered with a coverslip and studied under a fluorescent microscope (Olympus AX70, Japan.).

### Immunohistochemistry for S100

To identify and evaluate the purity of the Schwann cells, immunoassay of S100 was performed according to following procedure: The sections were washed 3 times in PBS for 6 minutes, incubated for 30 min in blocking solution [1× PBS + 0.1% Triton X +2% normal goat serum (NGS)], then incubated overnight at 4℃ with cell marker primary antibody (Rabbit Anti-S100, Dakocytomation, code No: Z0311). After rinsing 3 times in PBS, they were incubated with FITC conjugated secondary antibody in the dark at room temperature for 60 minutes. The sections were mounted onto gelatin-coated glass slides and were analyzed under a fluorescent microscope.

### Statistical analysis

The statistical comparisons between groups were carried out using repeated measures analysis of variance (ANOVA) followed with the tukey test for post hoc analysis.

Statistical analysis was performed using SPSS version 15. A p<0.05 was accepted to denote statistically significance and all data were presented as (mean ± SEM).

## Results

### Cell culture

BMSCs and SCs were cultured in DMEM medium. When they had grown to 80% confluency they were subcultured 3 and 4 times respectively (Fig 1A ,B).

### Histology findings

Four weeks after contusion injury observation of sections which stained with cresyl-violet indicates that vacuoles and cystic cavities of different sizes had formed at the site of injury. This cyst formation is due to the death of neurons, interneurons and glial cells after SCI.

### Immunohistochemistry findings

**Fig 2:**
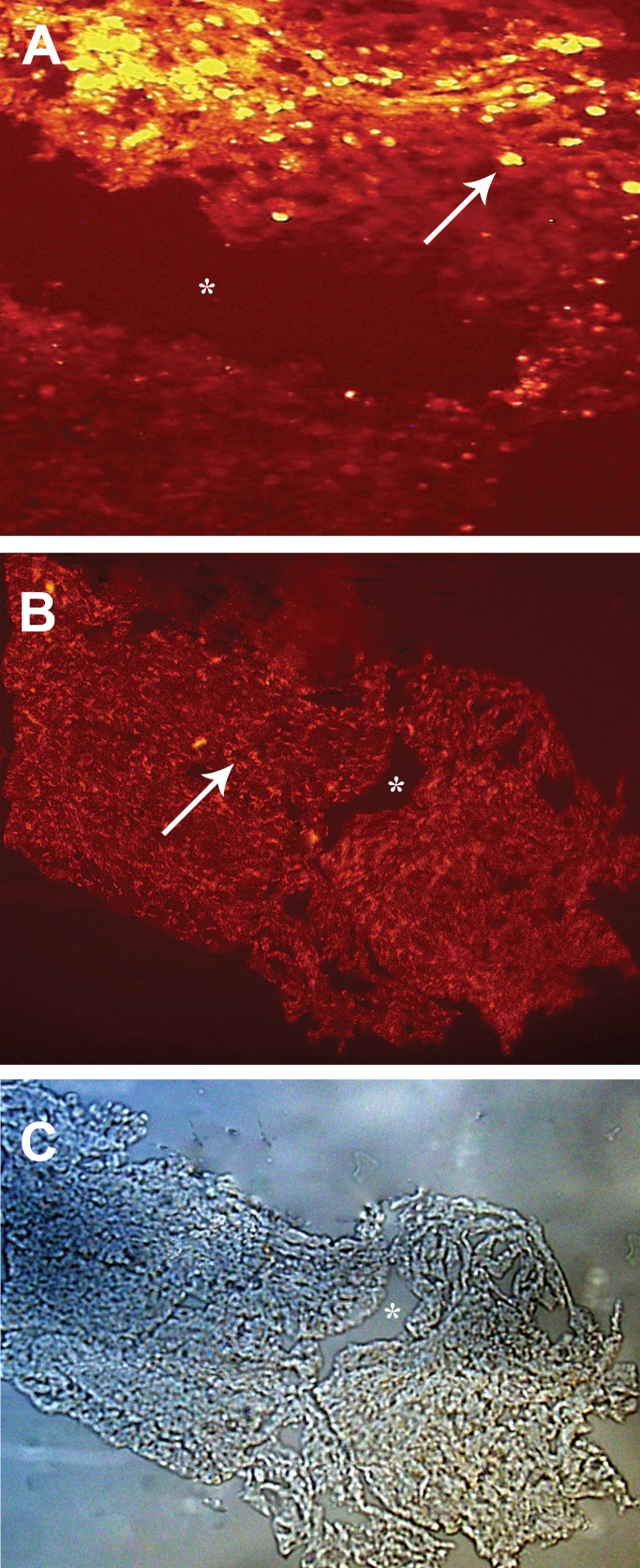
Immunohistochemical findings 8 weeks after transplantation of Bone marrow stromal cells and Schwann cells. A. DiI labeled Schwann cells survived and gathered around the injury site (×200). B. Brdu-labeled bone marrow stromal cells survived and gathered around the injury site (×100). C. Which is shown by phase contrast microscope (×100). Star indicates the site of injury. Arrows indicate the Bone marrow stromal cells and Schwann cells

Immunohistochemical findings showed that transplanted BMSCs and SCs survived at the site of injury 8 weeks after transplantation. Figure 2A shows DiI labeled Schwann cells, identified by fluorescent microscopy, and figure 2B shows Brdu- positive BMSCs survived and gathered around the injury site.

### Behavioral assessment (locomotor function)

Prior to SCI rats in all 3 experimental groups had a BBB score of 21 points. One day after SCI, the contused rats demonstrated considerable loss of hind limb locomotor function with no movement and BBB scores of 0-1 points.

On the following days the BBB score increased considerably in all groups. For example on the 14^th^ day post operation (DPO), mean ± SEM BBB scores were 8.12 ± 1.12 in the sham group, 10.25 ± 1.28 in the SC group, 10.62 ± 1.30 in the BMSC group and 10.75 ± 1.16 in the co-transplant group.

**Fig 3:**
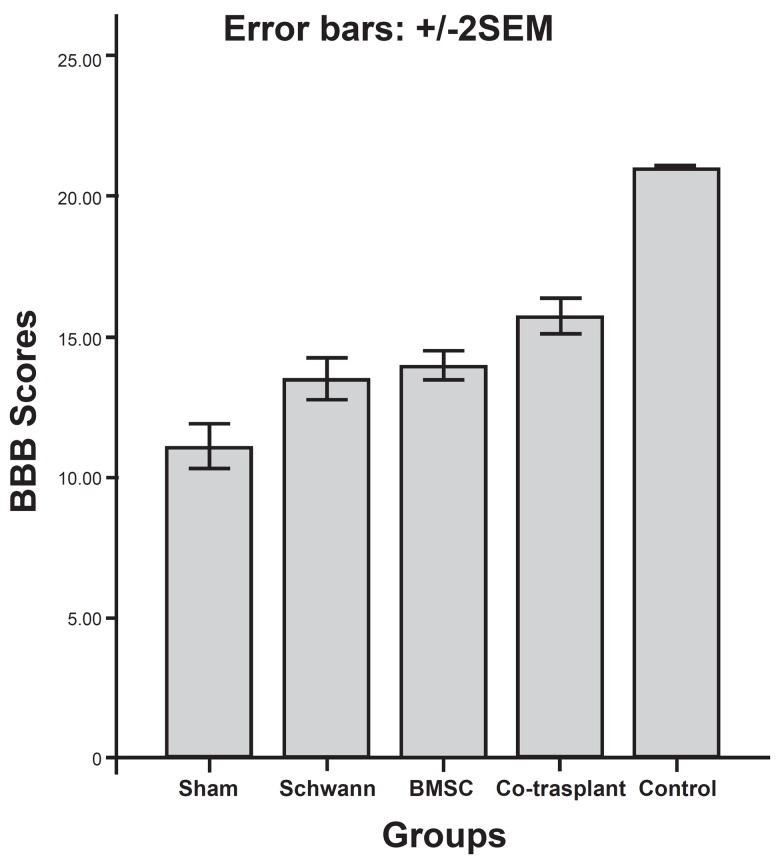
The bar graph representing the assessment of motor function recovery by BBB scores following cell transplantation. Eight weeks after cell transplantation there is significant difference between experimental and sham groups (p < 0.05).

**Fig 4:**
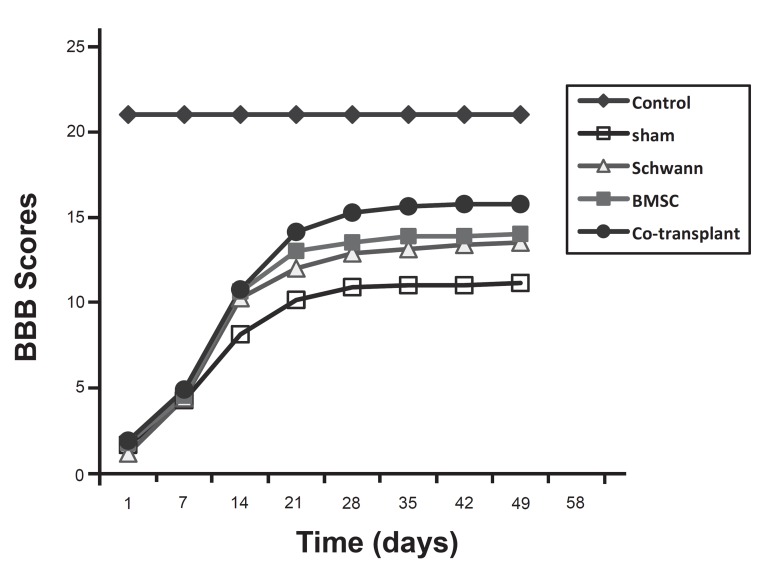
The BBB scores in different groups which have assessed weekly for 8 weeks post transplantation.

From weeks 3-8 animals in the SC, BMSC and co- transplant groups exhibited a progressive increase in their hindlimb movements in comparison to the sham group (p<0.05) (Figs 3 , 4).

In the 7^th^ and 8^th^ weeks walking skills among the rats in the co-transplant group improved significantly compared to the SC and BMSC groups. Moreover, the animals in the co-transplant group displayed consistent weight supported plantar stepping and demonstrated consistent fore-hindlimb coordination, whereas the animals in the SC and BMSC groups showed modest fore-hindlimb coordination.

A BBB score of 21 was obtained by animals in the control group during all stages of the study. The average BBB scores for animals in the sham, SC, BMSC and co-transplant groups were respectively: (11.12 ± 1.12), (13.5 ± 1.06), (14 ± 0.75) and (15.87 ± 0.83) in the 8th week (Figs 3 , 4) .

The statistical analysis revealed that there were significant differences between the experimental and control groups, between the experimental and sham groups and between the co-transplant and the SC or BMSC only groups (p<0.05). In contrast, the statistical difference between the SC and BMSC groups was not significant (p>0.05) (Figs 3 , 4).

### Behavioral assessment (mechanical Allodynia)

**Fig 5:**
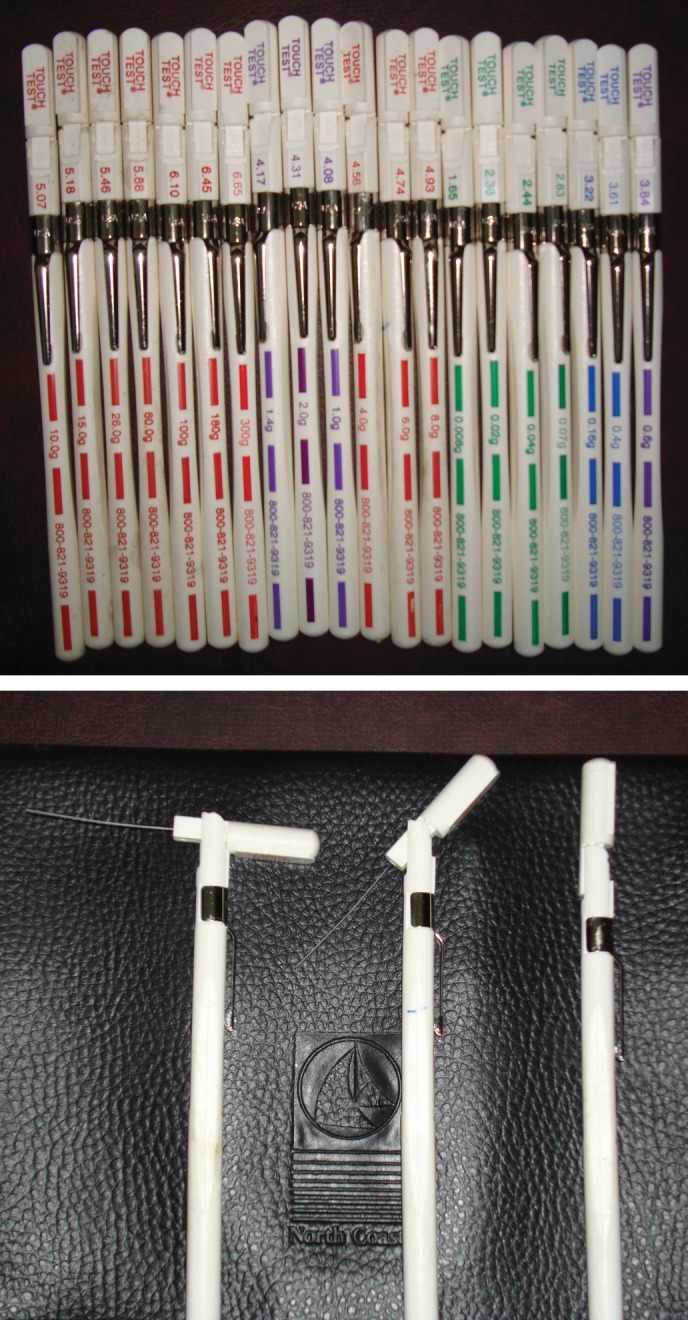
Von Frey Filaments for assessment of mean withdrawal threshold and mechanical allodynia

**Fig 6:**
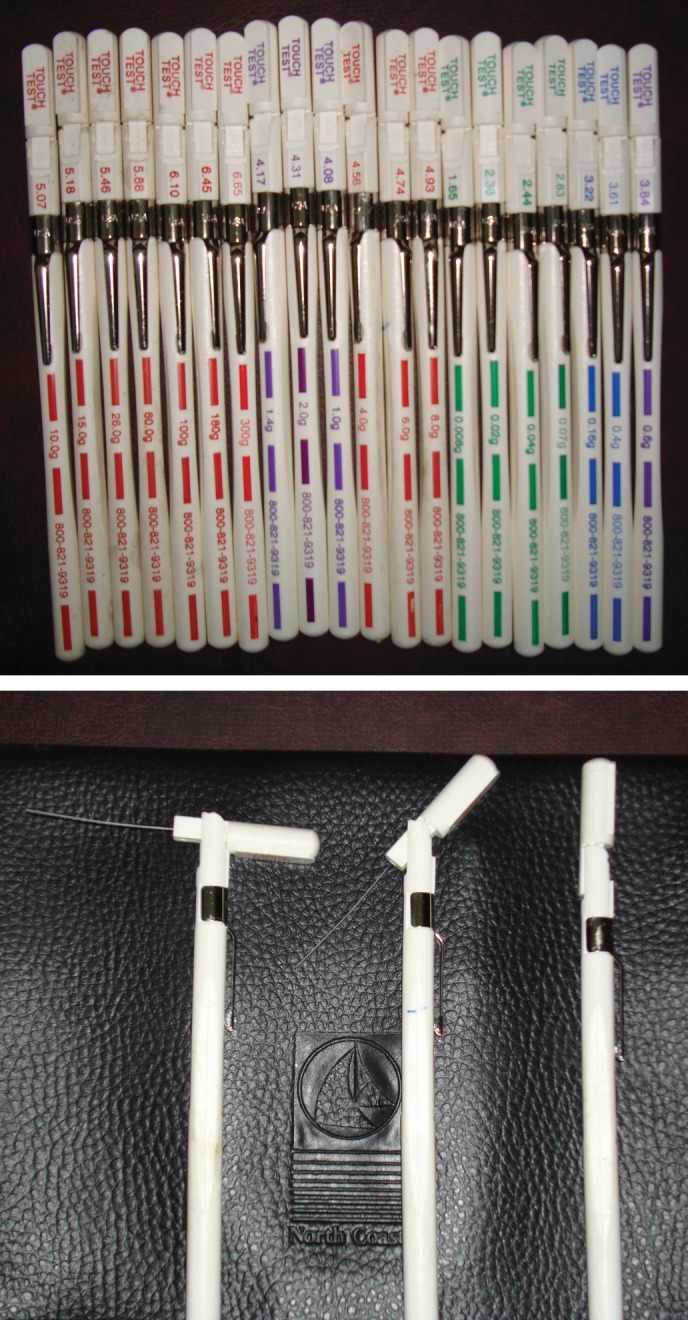
A. The line graph representing the assessment of mean withdrawal thresholds in different groups every week and for 8 weeks after cell transplantation. B. The graph showed that there is a significant difference in mean withdrawal threshold between experimental and sham groups

The Von Frey Hair Test was performed weekly for 8 weeks for the assessment of mechanical Allodynia (Figs 5 , 6). In the control group the mean withdrawal threshold was (60 ± 0 g), which was consistent over the 8 weeks. In the sham group the mean withdrawal threshold was (210 ± 19.64 g) in the first week, (110 ± 10 g) in the second week, (70 ± 6.54 g) in the third week, (55.75 ± 4.25g) in the fourth and fifth week and (51.5 ± 5.56 g) in the sixth, seventh and eighth week. At the end of eight week there was a significant difference between this group and the BMSC and SC only, and the co-transplant groups (p<0.05).

In the BMSC group the mean withdrawal threshold was (210± 19.64 g) in first week, (80.75±10.09 g) in the second week, (56.5 ± 8.25 g) in the third week, (41.63 ± 7.06 g) in the fourth week, (33.13 ± 6.01 g) in the fifth week, (27.5 ± 4.96 g) in the sixth and seventh weeks, and (21.88 ± 2.01 g) in the eighth week. At the end of the eighth week there was a significant difference between this group and the sham group (p<0.05) but no significant difference between this group and SC and the co-transplant groups (p> 0.05).

In SC group, the mean withdrawal threshold was (185 ± 19.18 g) in first week, (70.75 ± 9.47 g) in second week, (47.25 ± 6.22 g) in the third week, (41.63 ± 7.06 g) in the fourth week, (33.13 ± 6.01 g) in the fifth week, (30.38 ± 6.71 g) in the sixth week, (26.13 ± 5.20 g) in the seventh week and (20.63 ± 5.62 g) in the eighth week. At the end of the eighth week there was a significant difference between this group and the sham group (p< 0.05) but no significant difference between this group and the BMSC or co-transplant groups (p> 0.05).

In the co-transplant group the mean withdrawal threshold was (200 ± 23.90 g) in the first week, (75 ± 7.31 g) in the second week, (56.5 ± 8.25 g) in the third week, (47.25 ± 6.22 g) in the fourth week, (31.75 ± 6.39 g) in the fifth and sixth weeks, (30.38 ± 6.71 g) in the seventh week and (19.13 ± 2.01 g) in the eighth week. At the end of the eight week there was a significant difference between this group and the sham group (p<0.05) but no significant difference between this group and the BMSC or SC groups (p> 0.05).

## Discussion

The present study demonstrates that the co-transplantation of BMSCs and SCs into the injured spinal cord of rats can improve functional recovery and also leads to development of mechanical Allodynia. Our histological findings confirmed the contusion model of injury had worked in the expected manner. The SCs were harvested (Fig1A) and labeled with DiI; fluorescent microscopy showed DiI-labeled Schwann cells survived and gathered around the injury site and had an exogenous source (Fig2A). The BMSCs were cultured (Fig1B) and labeled with Brdu, immunohistochemistry showed that after 8 weeks, Brdu-positive BMSCs survived and gathered around the cavity center (Fig 2B).

Results from the behavioral assessment (locomotor function) showed that co-transplantation of BMSCs and SCs in an animal model of SCI can considerably enhance locomotor recovery and may prove a beneficial method of treatment for SCI (Figs 3, 4).

The BBB scores observed in our study in the BMSC and SC groups in the eighth week after injury are very similar to scores found in other studies. For instance, the scores for BMSC injected animals in the study of Ohta et al. (23) and Hofstetter et al. (24) are respectively: (13.87 ± 3.0), (13) and the score for SC injected animals in the study of Firouzi et al. (25) and Ban DX et al. (26) are respectively (13.5 ± 1.1) and (13.17 ± 0.71); that are very similar to our scores.

The data from the present study also indicates that co-transplantation of BMSCs & SCs into the injured spinal cord leads to an undesirable state named mechanical Allodynia. Spinal cord injury is often followed by debilitating neuropathic pain named Allodynia. Allodynia defined as increased sensitivity to normally innocuous stimuli, in other words Allodynia is a painful response to a nonpainful stimulus.

Several hypotheses have explained for etiology of allodynia: the study of Seung et al. showed that loss of spinal µ-opioid receptors after peripheral nerve injury result in allodynia (27).

The findings of the present study indicate that transplantation of BMSCs and SCs and a combination of these cells into the injured rat spinal cord significantly decrease the mean withdrawal threshold compared to a sham group which received only serum and any cell (p<0.05) (Fig 6A, B). In other words transplantation of these cells induces mechanical allodynia.

The findings of our study are similar to the studies of Hofstetter et al. (8) and Macias et al. (13). Hofstetter et al. (8) reported that grafting of adult NSCs into a rat thoracic spinal cord contusion injury improved motor recovery but also caused Allodynia. Macias et al. (13) have shown that transplantation of C17.2 NSCs into the injured spinal cord improved functional outcomes but also resulted in Allodynia.

The mean withdrawal threshold at the end of the eight week in the BMSC, SC and co-transplant groups were respectively (21.88 ± 2.01g), (20.63 ± 5.62 g) and (19.13 ± 2.01g) which indicates that the mean withdrawal threshold in the co-transplant group decreased further than in the BMSC and SC groups, suggesting that co-transplantation of BMSCs and SCs can induce a higher level of allodynia (Fig 6A, B).

It is possible that BMSCs and SCs transplanted into the spinal cord may differentiate mainly into astrocytes which provide NGF. NGF in turn can cause aberrant axonal sprouting in neurons of the dorsal horn which is associated with nociception and results in Allodynia. It is also possible that cotransplantation of BMSCs and SCs leads to more NGF production and more axonal sprouting in the dorsal horn which thus results in enhanced Allodynia.

The present study has also shown that although co-transplantation of BMSCs and SCs in an animal model of SCI can considerably enhance locomotor recovery. It also induces undesirable pain i.e. allodynia.

Some methods of reducing allodynia have been reported in a number of studies including: Transplantation of GABAergic cells (28); grafting cells which secrete serotonin, catecholamines and opioids, like the chromaffin cells of the adrenal medulla (29); administration of cyclooxygenase-2 (cox-2) inhibitor meloxicam (30); intrathecal administration of adenosine (31); intrathecal administration of lidocaine (32); administration of Gabapentin (33); spinal administration of δ opioid receptor agonists (34); applying methods which shift the differentiation of stem cells from an astrocytic line to an oligodendrocytic line (Transduction of stem cells with neurogenin 2 before transplantation) (8); or administration of Minocycline, which prevents glial cell (microglia and astroglia) activation and proinflammatory cytokine expression (35).

## Conclusion

Our findings have shown that co-transplantation of BMSCs and SCs may provide a powerful therapy for SCI. Further research is required for the development of combinatory treatment strategies in the future. The study has also shown that cotransplantation of these cells may lead to an increase in allodynia. For this reason future stem cell therapy programs should focus on combinational therapy using these cells at the same time as applying methods which can decrease the allodynia.
